# Driving With Hemianopia X: Effects of Cross Traffic on Gaze Behaviors and Pedestrian Responses at Intersections

**DOI:** 10.3389/fnhum.2022.938140

**Published:** 2022-07-11

**Authors:** Jing Xu, Vilte Baliutaviciute, Garrett Swan, Alex R. Bowers

**Affiliations:** ^1^Schepens Eye Research Institute of Massachusetts Eye and Ear, Boston, MA, United States; ^2^Department of Ophthalmology, Harvard Medical School, Boston, MA, United States; ^3^Envision Research Institute, Wichita, KS, United States

**Keywords:** driving, hemianopia, cross traffic, gaze behavior, visual field loss, intersection

## Abstract

**Purpose:**

We conducted a driving simulator study to investigate the effects of monitoring intersection cross traffic on gaze behaviors and responses to pedestrians by drivers with hemianopic field loss (HFL).

**Methods:**

Sixteen HFL and sixteen normal vision (NV) participants completed two drives in an urban environment. At 30 intersections, a pedestrian ran across the road when the participant entered the intersection, requiring a braking response to avoid a collision. Intersections with these pedestrian events had either (1) no cross traffic, (2) one approaching car from the side opposite the pedestrian location, or (3) two approaching cars, one from each side at the same time.

**Results:**

Overall, HFL drivers made more (*p* < 0.001) and larger (*p* = 0.016) blind- than seeing-side scans and looked at the majority (>80%) of cross-traffic on both the blind and seeing sides. They made more numerous and larger gaze scans (*p* < 0.001) when they fixated cars on both sides (compared to one or no cars) and had lower rates of unsafe responses to blind- but not seeing-side pedestrians (interaction, *p* = 0.037). They were more likely to demonstrate compensatory blind-side fixation behaviors (faster time to fixate and longer fixation durations) when there was no car on the seeing side. Fixation behaviors and unsafe response rates were most similar to those of NV drivers when cars were fixated on both sides.

**Conclusion:**

For HFL participants, making more scans, larger scans and safer responses to pedestrians crossing from the blind side were associated with looking at cross traffic from both directions. Thus, cross traffic might serve as a reminder to scan and provide a reference point to guide blind-side scanning of drivers with HFL. Proactively checking for cross-traffic cars from both sides could be an important safety practice for drivers with HFL.

## Introduction

Hemianopic field loss (HFL) is the loss of half the field of vision on the same side in both eyes, commonly caused by stroke and traumatic brain injury. People with HFL may be permitted to drive in some jurisdictions in the United States (Peli, 2002), some provinces in Canada (Yazdan-Ashoori and Ten Hove, 2010; Dow, 2011), and in some other countries such as Belgium (Bowers et al., 2012), the Netherlands (Tant et al., 2002; de Haan et al., 2014), and the United Kingdom (DVLA Drivers Medical Group, 2011). However, the hemifield loss may cause difficulties when driving especially in complicated road situations, such as at intersections.

When approaching and driving through an intersection, drivers must be mindful of other road users, including cross traffic and pedestrians. Failing to notice and respond to other road users may result in a collision. According to the Federal Highway Administration, in 2019, traffic at intersections accounted for 29% of all vehicle-involved fatal crashes (National Highway Traffic Safety Administration [NHTSA], 2020). Intersections are challenging for drivers with HFL because a wide field of view needs to be scanned to check for hazards. Individuals with HFL lack peripheral vision on the side of the hemianopia (the blind side), and therefore have to scan sufficiently far toward that side (at least as far as the object of interest), in order to detect other road users on that side. Not surprisingly, in prior driving simulator studies (Bowers et al., 2009, 2014; Papageorgiou et al., 2012; Swan et al., 2021b), some individuals with HFL exhibited blind-side scanning deficits resulting in impaired detection of blind-side hazards at intersections.

There are many factors that could affect scanning at intersections such as the intersection configuration (Bowers et al., 2014; Yan et al., 2018), the type of traffic control device (Lemonnier et al., 2015; Li et al., 2019; Savage et al., 2021), and the presence of cross traffic or different traffic densities (Rahimi et al., 1990; Bao and Boyle, 2009; Dukic and Broberg, 2012; Werneke and Vollrath, 2012, 2014; Zhang et al., 2012; Lemonnier et al., 2015; Kazazi et al., 2016; Feng et al., 2018; Thompson and Sabik, 2018). In studies of normally sighted drivers, participants exhibited more frequent scans and fewer fixations on the road ahead (more fixations on moving objects) when there were other road users or more traffic at intersections (Rahimi et al., 1990; Werneke and Vollrath, 2012, 2014; Zhang et al., 2012; Kazazi et al., 2016). In a study of drivers with HFL, Papageorgiou et al. (2012) found that participants who performed well in a simulated intersection collision avoidance task scanned more to the blind side and fixated more on vehicles and less on the travel direction and road than participants who performed less well. However, the task did not require any driving maneuvers. Therefore, in the current study we used a driving simulator to evaluate the effects of intersection cross traffic on gaze and driving behaviors of individuals with HFL and age-similar controls with normal vision (NV) in a driving task where participants had full control of both vehicle speed and steering. The scenarios included a crossing pedestrian at the exit to the intersection so that we could examine the effects that monitoring cross traffic might have on the safety of driving responses (braking) to the pedestrian.

We were interested in investigating important questions, not previously addressed, about the effects that monitoring cross traffic might have on scanning behaviors of drivers with HFL and their responses to crossing pedestrians. On the one hand, drivers with HFL might prioritize scanning to look at cross traffic approaching from the blind side and be slower to notice or fail to notice cars and pedestrians approaching from the seeing side. On the other hand, looking at cross traffic on the seeing side might take attention away from the blind side and reduce scanning to the blind side, resulting in detection failures for cars and pedestrians from the blind side. Thus, we designed scenarios where the cross traffic approached from the blind side only, the seeing side only, or both sides simultaneously. We expected that gaze behaviors would differ between scenarios with and without a cross-traffic car on the seeing side. Specifically, we hypothesized that the presence of a seeing-side car would increase the time taken to first fixate cross traffic on the blind side and would reduce the amount of time spent looking at cross traffic on that side. In addition, we expected that scanning and fixating on cross-traffic cars would have positive benefits for the safety of responses to pedestrians crossing the road at the exit of the intersection because the full width of the intersection would have to be scanned to fixate on the cross traffic. We hypothesized that the rate of unsafe responses to pedestrians on the blind side would be highest when there were no cross-traffic fixations. Finally, given that drivers with HFL may prioritize scanning to the blind side at the expense of scanning to the seeing side, we also compared seeing-side scanning behaviors and response safety of drivers with HFL to performance of drivers with NV.

## Materials and Methods

### Participants

Sixteen participants (Table 1) with hemianopic field loss (HFL; 11 with Left HFL, 5 with Right HFL) were recruited from a database of individuals who participated in prior studies at the Schepens Eye Research Institute. They all had visual acuity of at least 20/40 (the minimum visual acuity for an unrestricted license in Massachusetts) and no visual neglect [as measured by the Bells test (Vanier et al., 1990) and Schenkenberg line bisection test (Schenkenberg et al., 1980)]. They also completed the Montreal Cognitive Assessment (MoCA, Nasreddine et al., 2005) and a questionnaire addressing demographic information, ocular history, and driving experience. HFL participants’ visual fields were measured using a Goldmann perimeter (kinetic V4e target). Thirteen individuals had complete homonymous hemianopia, two had incomplete hemianopia with residual vision in the upper part of the superior area of the field on the side of the hemianopia but complete field loss in the area where pedestrians and cross traffic vehicles appeared, and one had incomplete upper quadrantanopia. Visual inspection of the data for the three participants who did not have complete hemianopia indicated that their driving performance, gaze scanning and gaze fixation behaviors fell within the range of the other participants with complete hemianopia; therefore their data were included in analyses.

**TABLE 1:** Characteristics of the 32 study participants in the analyses.

Characteristic	HFL group (n = 16)	NV group (n = 16)
Age, years, median (IQR)	53.5 (38.5, 61)	50 (39, 66)
Male, n (%)	13 (81%)	10 (63%)
Binocular VA, logMAR, median (IQR)	–0.05 (–0.08, 0)	–0.07 (–0.11, –0.03)
MoCA score, median (IQR)	28 (25, 29)*	NA
Right HFL, n (%)	5 (31%)	NA
Years since onset, median (IQR)	3.5 (2, 9.5)	NA
Hemianopia caused by stroke, n (%)	10 (63%)	NA
Current driver, n (%)	3 (19%)	14 (88%)
Total years driving experience, median (IQR)	27 (7, 37)	32 (23, 50)

*IQR, interquartile range; VA, visual acuity; logMAR, logarithm of the minimum angle of resolution; MoCA, Montreal Cognitive Assessment; HFL, hemianopic field loss; NV, normal vision.*

*Data are missing for two participants, but they had sufficient cognitive functioning to participate.

Three HFL participants were current drivers, 13 were former drivers with a median 20 (IQR: 3–36) years driving experience who had stopped driving on median 5 (IQR: 2–15) years ago. The majority of HFL participants were not active drivers at the time of the study because a horizontal visual field extent of at least 120° is required for driving licensure in Massachusetts where the study was conducted. However, all of the former drivers were very keen to drive if it were possible, and this was a strong motivating factor for participating in the driving simulator study. None of the HFL participants experienced simulator sickness, thus all 16 were included in analyses.

In addition, 20 current drivers with no visual impairment (normal vision, NV) were recruited to provide age-similar comparison data. Four NV participants did not finish the study because of simulator sickness, thus data for 16 NV participants (Table 1) were included in analyses. Two NV participants were not current drivers but had extensive prior driving experience (>41 years). The study was conducted in accordance with the tenets of the Declaration of Helsinki and approved by the institutional review board (IRB) of Massachusetts Eye and Ear. Written informed consent was obtained from all participants after a full explanation of the study procedures.

### Materials

#### Apparatus

Participants drove in a fixed-base driving simulator comprising a customized simulator frame, a standard car seat, a Fanatec ClubSport Wheel Base V2 steering wheel, and a ClubSport V3 pedal set with automatic transmission (Fanatec, Landshut, Germany). The seat, wheel, and pedal placement could be adjusted to provide a comfortable driving posture for each participant. Three Samsung 34-inch Ultra WQHD (wide quad high definition) screens were fixed to the frame, providing 165.5° horizontal by 26.5° vertical field of view. A Tobii 4C eye tracker (Tobii 4C, Danderyd, Sweden) was mounted below the center screen and used to track the driver’s gaze position on that screen (about a 55° horizontal field of view) at 90 Hz.

#### Driving Simulation Environment

The driving simulation software and virtual environment were developed in-house using Unity 3D (Unity Technologies, San Francisco, CA). We created a customized, populated city environment, using high fidelity 3D models and animations to simulate pedestrians, crowds, cross traffic, and other vehicles on the road. A total of 21 vehicles with different colors and models and 31 different life-size human models were used. The pedestrians and crowds were programmed to move at average human walking and jogging speeds (1.2 − 1.6 m/s). For the driver’s vehicle, high-fidelity vehicle physics were applied based on a typical four-door sedan. The software recorded information about the driver’s vehicle (speed, location, the status of controls), all other programmed entities (cross traffic, pedestrians, etc.) in the virtual environment, and gaze tracking data at a sampling rate of 50 Hz. The eye tracker data were down sampled from 90 to 50 Hz using the Tobii Eye Tracking Unity SDK (developer version 4.0.3).

**FIGURE 1:**
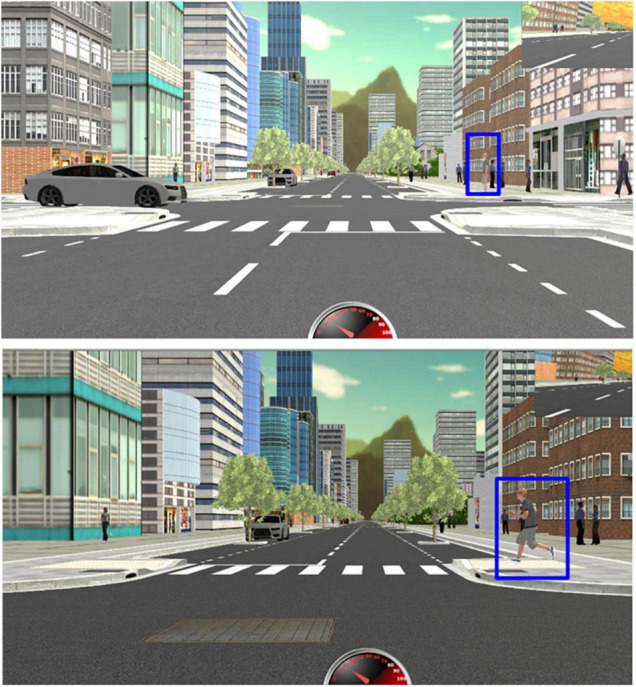
Screenshots of a critical event (Scenario 2) at a four-way intersection viewed from the perspective of the driver (only the view on the center monitor is shown). ***Top***—when the driver approached the intersection, a gray car approached the intersection from the left side, and a pedestrian (marked with a blue rectangle) was standing on the right on the far side of the intersection. ***Bottom***—when the driver entered the intersection, the pedestrian started running across the road at the zebra-marked crosswalk, requiring the driver to brake to avoid a collision.

Two drives along different routes were created within the city environment, each about 5 km in length, with pedestrian crowds and traffic throughout the routes and a mixture of parked cars and trees in parking lanes (Figure 1). There were 45 intersections (95% four-way and 5% T intersections) in each drive, including intersections with stop signs and no signage. The city environment simulated an urban area (Figure 1); road geometry, sidewalk, and crosswalk designs followed American Association of State Highway and Transportation Officials (AASHTO) “Policy on Geometric Design of Highways and Streets” guidelines (American Association of State Highway and Transportation Officials [AASHTO], 2018). The posted speed limit was 35 mph (56.3 km/h). A speed cap prevented participants from exceeding that speed. Pre-recorded GPS audio cues (e.g., “turn right at next intersection”) were used to direct participants along the pre-determined routes.

#### Intersection Events

**FIGURE 2:**
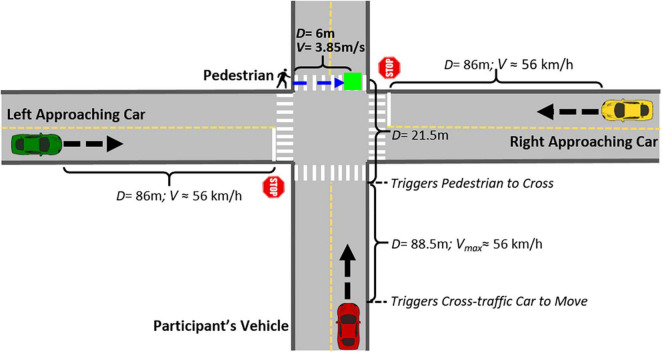
Schematic representation of an intersection event for a scenario with a cross-traffic car approaching from the left and the right (*Scenario 3* in Figure 3). The green square represents the collision point between the participant’s vehicle and the pedestrian that starts approaching from the left after the participant’s vehicle enters the intersection. (D, distance and V, velocity).

Critical event scenarios were scripted at 15 four-way intersections in each drive at which the intersection maneuver for the participants was to go straight ahead. These intersections did not have any signage on the driver’s approach but had stop signs on the cross street (Figures 1, 2). The other 30 non-critical intersections in each drive included either stop signs or no signs on the participant’s approach and a variety of intersection maneuvers (go straight, left and right turns).

**FIGURE 3:**
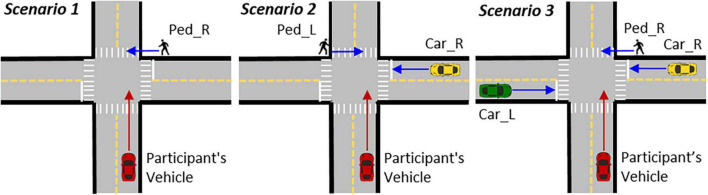
Schematic of each of the three types of intersection scenarios. Each scenario included a pedestrian on either the left or right side. *Scenario 1* **(left)** had no cross traffic and a pedestrian on either the left or right, shown here with a pedestrian on the right (Ped_R). *Scenario 2* **(middle)** included a cross-traffic vehicle approaching from one side only with a pedestrian on the opposite side, shown here with a car from the right (Car_R) and a pedestrian on the left (Ped_L). *Scenario 3* **(right)** included cross traffic from both the left (Car_L) and right (Car_R) sides at the same time with a pedestrian on either the left or right, shown here with a pedestrian on the right (Ped_R).

Three types of critical event scenarios were developed (Figure 3), with 10 of each type across the two drives: ***Scenario 1***—intersection without cross traffic; ***Scenario 2***—intersection with one car approaching from either the left (*n* = 5) or right (*n* = 5); and ***Scenario 3***—intersection with two cars approaching at the same time, one from the left and one from the right side. Cross traffic was triggered to appear on the cross street when the participant’s vehicle was 88.5 m from the entrance to the intersection. The cross traffic approached the intersection with a mean speed of 35 mph (about 56.3 km/h). The stop signs on the cross streets indicated that the cross-traffic cars had to stop and yield to the participant’s vehicle. Therefore, the cross traffic came to a complete stop before entering the intersection.

In the current study we simulated a real-life situation where the driver had to respond (by braking) to avoid a potential collision with a pedestrian crossing the road. Since we wanted to investigate the effects of monitoring cross-traffic on responses to crossing pedestrians, for each critical event scenario, a pedestrian was placed on the sidewalk on the far side (exit side) of the intersection 6 m to either the left or the right of the potential collision point with the participant’s vehicle (Figures 2, 3). For each of the three types of scenarios, there were five events with the pedestrian on the right side and five events with pedestrians on the left side. The pedestrian was programmed to stand at that location during the entire drive, and then suddenly run across the crosswalk at 3.85 m/s (about 8.6 mph) when the participant’s car entered the intersection. In *Scenario 2*, where there was only one cross-traffic car from either the left or right, the car always approached from the side opposite the pedestrian. To prevent any discomfort that may have been caused by actually colliding with pedestrians, the pedestrian was programmed to disappear immediately after the front of the car touched the pedestrian.

To decrease anticipation of critical events, nine distraction events were programmed at non-critical intersections in each drive. These events included: intersections with cross traffic from one or both sides, which would stop before the stop line, without a pedestrian; intersections with cross traffic from one or both sides, which would cross the intersection without stopping, with or without a pedestrian; intersections without any cross traffic, but with one or multiple pedestrians standing around the crosswalk area at the far side of the intersection; intersections without any cross traffic, but with a pedestrian standing around or crossing the crosswalk area at the near side of the intersection. Besides these distraction events, there were also other cars and crowds (standing, walking, jogging people) scattered throughout the city environment to simulate a real-world environment.

### Procedures

The study was completed in one visit for each participant. After providing informed consent, participants completed vision measurements, screening tests, and vision history and driving experience questionnaires. If eligible, participants would proceed with the drives. There were four drives; two practice drives and two test drives. Participants began with the practice drives to acclimate to the simulator. The first practice drive was set in a city environment similar to the one in the experimental drives, but with no traffic or pedestrians. Participants practiced listening to GPS instructions, braking, accelerating, and making turns. The second practice drive included all types of intersection event scenarios in the experimental drives for participants to practice responding to cross traffic and pedestrians in the virtual world. After the practice drives, participants’ gaze was calibrated with Tobii’s 6-point calibration software and validated using custom, 9-point verification software in Unity. If overall verification accuracy was worse than 2°, then the calibration procedure was repeated, and accuracy verified once more. Then the participant completed the two experimental drives, with the order counterbalanced across participants. Participants were instructed to drive at a speed limit of 35 mph and to respond to traffic as they normally would in real life, obey traffic rules, follow GPS navigation, and avoid collisions. The whole study took about 2 h including practice and experimental drives.

### Data Processing

Data from the 15 critical-event intersections in each drive were processed and analyzed. Data from the other intersections were not analyzed.

#### Quantifying Gaze Scanning Behaviors

Participants’ scanning behaviors were quantified in terms of the number, direction and magnitude of gaze scans on approach to the intersection (from the time when the participant’s car was 88.5 m from the intersection to the time when the car entered the intersection). Gaze scans were defined as the entire series of lateral gaze movements (i.e., saccades) that typically started from the straight-ahead position (0°) and ended in the periphery to the left or to the right. Gaze scans were marked automatically using a custom algorithm (Swan et al., 2021a). Only gaze scans larger than 4° were included. See Supplementary Figure 1 for examples of gaze scanning on approach to an intersection.

#### Categorizing Events by the Number of Cross-Traffic Cars Fixated

To address our questions about the effects of fixating on cross-traffic cars on either the blind side, the seeing side, or both sides, each critical event was categorized by the number of cross-traffic cars that were fixated. There were three categories: did not fixate on cross traffic (*NoFix)*, only fixated on one cross-traffic car (*Fix_OneCar*), and fixated on both cross-traffic cars (*Fix_BothCar*). Thus, *NoFix* included all events for *Scenario 1*, as well as those events for *Scenarios 2* and *3* where none of the cross traffic was fixated. *Fix_OneCar* included events from *Scenarios 2* and *3* where participants fixated on a single cross-traffic car and *Fix_BothCar* included only events from *Scenario 3* where participants fixated on both cross-traffic cars. See Supplementary Figure 1 for examples of different intersection scenarios and events with different numbers of cars fixated.

Of the 480 possible events for those with HFL, 205 events were categorized as *NoFix*, 198 events were categorized as *Fix_OneCar*, and 77 events were categorized as *Fix_BothCar*. For the NV group, there were 480 events in total and 15 event data was removed due to data loss. Of these 465 events, 184 events were categorized as *NoFix*, 205 were categorized as *Fix_OneCar*, and 76 were categorized as *Fix_BothCar*.

#### Quantifying Gaze Fixations on Cross-Traffic Cars

Fixations were defined as gaze that fell within the bounding box area (i.e., a boundary of 1° around the object) for durations greater than 120 ms and with dispersion less than 1°(Salvucci and Goldberg, 2000). The “time to the first fixation” was defined as the time from when the cross traffic was triggered to when the participant made the first fixation on the cross traffic. “Fixation duration” was computed as the total duration of time for which gaze was on the cross traffic; if a participant made multiple fixations, then it was the total time summed across all fixations.

#### Quantifying Gaze Fixations on Pedestrians

Gaze fixations on pedestrians were defined in the same way as gaze fixations on cross-traffic cars, except that the time to the first fixation was defined as the time from when the cross traffic was triggered to when the participant made the first fixation on the pedestrian, and fixation duration was computed as the total duration of time for which gaze was on the pedestrian.

#### Quantifying Driving Response to Pedestrians

Participants’ driving responses to the crossing pedestrian were categorized as safe or unsafe based on the post encroachment time (PET), a measure that is commonly used to quantify pedestrian-vehicle interactions (Zangenehpour et al., 2016; Johnsson et al., 2018). It is defined as the time difference between the first road user leaving the collision zone and the second road user entering it (Allen et al., 1978), providing a measure of the safety margin or the extent to which the two road users (the participant’s car and crossing pedestrian in this study) miss each other. The smaller the post encroachment time, the more unsafe the situation. For the current study, the post encroachment time was calculated per frame from the time when the pedestrian started moving until the participant’s car passed the pedestrian. If the post encroachment time was ever less than 1 s, the event was classified as “unsafe” [since that is considered a dangerous situation in real-world traffic conflicts (Hupfer, 1997)]; otherwise, it was “safe.” When the participant’s car and crossing pedestrian came into contact with each other, the event was categorized as a “collision.”

### Statistical Analyses

The statistical analyses were performed using R (Version 1.1.463). A significance level of *p* = 0.05 was applied for all statistical tests. In preliminary analyses, no significant differences were found between the left and right sides for all gaze scanning, gaze fixation, and safety response measures of NV participants. Therefore, for the main analyses we collapsed the left and right sides together for NV participants and compared the average of the left- and right-side NV performance with the blind side or seeing side for HFL participants.

Linear Mixed Models (LMM) were utilized for continuous variables that were normally or close to normally distributed (time to first fixation and fixation duration on cross traffic or pedestrian, median scan magnitude). For non-continuous measures (number of gaze scans, unsafe responses), a General Linear Mixed Effect Model (GLM) was applied. In each model there were two fixed factors, either side [scan side or cross traffic/pedestrian side (*HFL_blind side*, *HFL_seeing side*, *NV combined right and left sides*)], and the number of cars fixated (*NoFix*, *Fix_OneCar*, *Fix_BothCar*) as well as their interactions. Unsafe response rate was the only measure for which there was a significant interaction between these fixed factors. Therefore, the effects of the number of cars fixated are reported separately for side (*HFL blind side*, *HFL seeing side* and *NV combined right and left sides*) for unsafe response rates but collapsed across this factor for all other measures. In all the LMM and GLM analyses, the event number and participant ID were included as random factors to account for variance between the different intersection configurations for each event and the variability from individual differences; the mean (*M*), standardized coefficients (β), standard error (*SE*), *t*-value (*t*) or *z*-score (*z*), and *p*-value (*p*) are reported. Wilcoxon tests were used to analyze variables “fixation rate on cross traffic or pedestrians” and “proportion of first scan direction” and the median (*Mdn*) and *p*-value are reported.

## Results

### Gaze Scanning When Approaching Intersections

The first set of analyses evaluated the effect of the subject group (HFL and NV), number of cross-traffic cars fixated (No fixation, only fixated on one car, fixations on both cars), and scan side (blind side or seeing side for HFL drivers and left and right sides combined for NV drivers) on gaze scanning behavior when approaching an intersection. The direction of the first scan, the number of scans per intersection event, and the median magnitudes of all gaze scans were used to assess gaze scanning behavior.

#### Direction of First Scan

For the HFL group, a significantly greater proportion of first scans were made to the blind than the seeing side (*Mdn*: 65% vs. 35%; *p* = 0.004). In contrast, there was no significant difference in first scan directions between left and right sides for the NV group (*Mdn*: 52% vs. 48%; *p* = 0.07).

#### Number of Scans

When approaching the intersection, HFL participants made significantly more scans to the blind side than the seeing side (*M*: 5.1 vs. 3.8; β = –0.29, *SE* = 0.03, *z* = –8.79, *p* < 0.001), but fewer scans to the seeing side than the NV participants did to either the left or right side (*M*: 3.8 vs. 4.6; β = 0.19, *SE* = 0.08, *z* = 2.37, *p* = 0.02). Greater numbers of scans occurred when participants fixated a car on both sides than a car on only one side (*M*: 5.2 vs. 4.5; β = –0.09, *SE* = 0.03, *z* = –2.52, *p* = 0.01), or did not fixate on any cars (*M*: 5.2 vs. 4.3; β = 0.15, *SE* = 0.04, *z* = 4.08, *p* < 0.001).

#### Median Gaze Scan Magnitudes

HFL participants made significantly larger gaze scans to the blind side than the seeing side (M: 9.6° vs. 9.05°; β = –0.03, *SE* = 0.01, *t* = –2.40, *p* = 0.016) and their seeing side scans were significantly larger than the scans of NV participants (M: 9.05° vs. 8.1°; β = –0.04, *SE* = 0.02, *t* = –2.22, *p* = 0.03). Larger gaze scans occurred when participants fixated a car on both sides compared to when they did not fixate on any car (M: 9.2° vs. 8.1°; β = 0.06, *SE* = 0.01, *t* = 4.11, *p* < 0.001), but did not differ between when they fixated on both cars and one car (M: 9.2° vs. 8.9°; β = –0.02, *SE* = 0.01, *t* = –1.29, *p* = 0.20).

### Gaze Fixation on Cross Traffic

The second set of analyses evaluated the effect of the subject group and the side from which cross-traffic cars approached (blind side or seeing side for HFL drivers and left and right sides combined for NV drivers) on cross-traffic car fixation behavior. We specifically assessed the proportion of cross-traffic cars fixated, time to first fixation and fixation duration on cross-traffic cars. Due to different numbers of cross-traffic cars in Scenarios 2 and 3, data from these scenarios were analyzed separately.

#### Scenario 2

**FIGURE 4:**
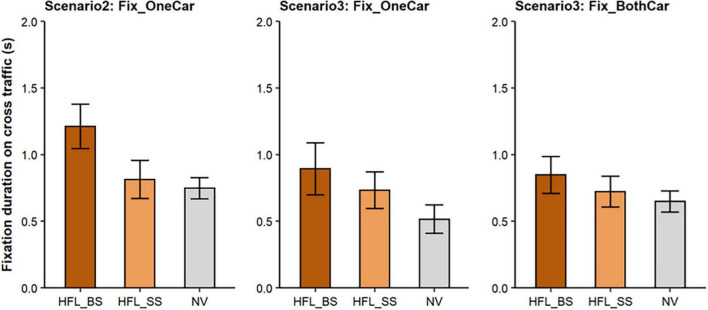
Mean fixation durations on cross-traffic cars on the HFL group’s blind side (HFL_BS), seeing side (HFL_SS), and for the NV group (average across left and right sides). ***Left-*** Scenario 2 (cross traffic from one side only): in cases when participants fixated on the cross traffic from the one side. ***Middle-*** Scenario 3 (cross traffic from both sides): in cases when participants fixated only on cross traffic approaching from one side. ***Right*-** Scenario 3: in cases when participants fixated on cross traffic approaching from both sides. Error bars represent SEM.

In Scenario 2, there was a cross-traffic car from one side only. The fixation rates on this car were significantly lower for the HFL group compared to the NV group (*Mdn:* 80% vs. 90%; *p* = 0.04) but did not differ between blind side and seeing-side cars for the HFL group (*Mdn:* 90% vs. 80%; *p* = 0.94). The side from which the car approached had no significant effect on the time to the first fixation (*M*: HFL blind side 5.71 s vs. HFL seeing side 5.55 s, β = –0.07, *SE* = 0.11, *t* = –0.66, *p* = 0.51; *M*: HFL seeing side 5.55 s vs. NV 5.68 s, β = 0.04, *SE* = 0.13, *t* = 0.25, *p* = 0.81). However, HFL participants had significantly longer fixation durations on blind-side than seeing-side cars (*M*: 1.21 s vs. 0.81 s; β = –0.02, *SE* = 0.05, *t* = –4.67, *p* < 0.001; Figure 4, left), but fixation durations for seeing-side cars did not differ from fixation durations of NV participants (*M*: 0.81 s vs. 0.75 s; β = 0.001, *SE* = 0.06, *t* = –0.02, *p* = 0.99).

#### Scenario 3

In Scenario 3, cross-traffic cars approached from both sides. Participants fixated on at least one car in the majority of cases with no significant difference between the HFL and NV groups (*Mdn:* 100% vs. 95%; *p* = 0.21). Fixations on both cars, one car, and no cars occurred in 48, 48, and 4% of events, respectively, for the HFL group, and 50, 41, and 9% of events, respectively, for the NV group. When HFL participants only fixated on one car, they were as likely to look at the car on their blind side as the car on their seeing side (*Mdn:* 50% vs. 50%, *p* = 0.80).

***When participants fixated on only one car***, HFL participants took significantly longer to fixate on blind-side than seeing-side cars (*M:* 6.11 s vs. 5.60 s; β = –0.54, *SE* = 0.24, *t* = –2.21, *p* = 0.03) but fixation durations on blind-side cars did not differ from those on seeing-side cars (*M:* 0.89 s vs. 0.73 s; β = –0.22, *SE* = 0.17, *t* = –0.1.32, *p* = 0.20). In contrast, first fixation times for seeing-side cars did not differ from those of NV participants (*M:* 5.60 s vs. 5.80 s; β = 0.16, *SE* = 0.22, *t* = 0.73, *p* = 0.47) but seeing-side fixation durations were significantly longer than those of NV participants (*M:* 0.73 s vs. 0.52 s; β = –0.44, *SE* = 0.14, *t* = –3.16, *p* = 0.006; Figure 4, middle).

***When participants fixated on cars from both directions***, the side of the approaching car had no significant effect on first fixation times (*M*: HFL blind side 5.32 s vs. HFL seeing side 5.58 s, β = 0.24, *SE* = 0.17, *t* = 1.43, *p* = 0.15; *M*: HFL seeing side 5.58 s vs. NV 5.38 s, β = –0.22, *SE* = 0.16, *t* = –1.40, *p* = 0.17) and no significant effect on fixation durations (M: HFL blind side 0.85 s vs. HFL seeing side 0.72 s, β = –0.09, *SE* = 0.05, *t* = –1.64, *p* = 0.10; M: HFL seeing side 0.72 s vs. NV 0.65 s, β = –0.04, *SE* = 0.06, *t* = –0.65, *p* = 0.52; Figure 4, right).

### Gaze Fixation on Pedestrians

In the third set of analyses, we assessed the effect of subject group, number of cross-traffic cars fixated, and pedestrian location (blind side or seeing side for HFL drivers and left and right sides combined for NV drivers) on drivers’ gaze fixation behavior on pedestrians. We specifically assessed fixation rate, time to first fixation and fixation duration on pedestrians.

Participants looked at the majority of pedestrians when approaching intersections, with no difference in the overall fixation rate between the HFL and NV groups (*Mdn:* 83% vs. 83%; *p* = 0.48). However, HFL participants had significantly lower fixation rates for pedestrians that appeared on their blind than their seeing side (*Mdn:* 80% vs. 93%; *p* = 0.02).

**FIGURE 5:**
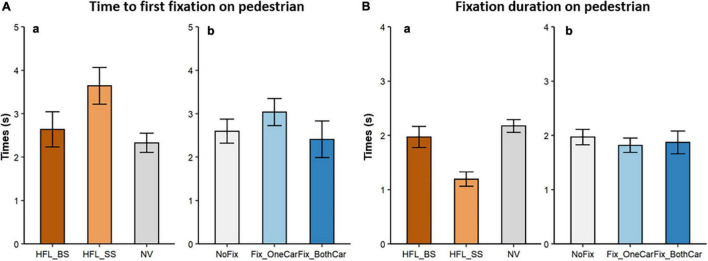
Mean time to first fixation on pedestrians **(A)** and mean fixation durations on pedestrians **(B)**. **(a)** comparison between pedestrian approaching side for HFL (blind and seeing side) and NV groups. **(b)** comparison among the three cross traffic fixation situations [no fixations on cross traffic (NoFix), fixate on one side of cross-traffic (Fix_OneCar), fixate on both sides of cross traffic (Fix_BothCar)]. Error bars represent SEM.

For HFL participants, first-fixation times were significantly ***shorter*** *(faster)* for blind-side than seeing-side pedestrians [*M:* 2.64 s vs. 3.64 s; β = 0.21, *SE* = 0.05, *t* = 3.99, *p* < 0.001; Figure 5, left (a)] but fixation durations were significantly ***longer*** [*M:* 1.97 s vs. 1.19 s; β = –0.32, *SE* = 0.05, *t* = –7.25, *p* < 0.001; Figure 5, right (a)]. In contrast, seeing-side first-fixation times were significantly ***longer*** than those of NV participants [*M:* 3.64 s vs. 2.33 s; β = –0.20, *SE* = 0.06, *t* = –3.16, *p* = 0.003; Figure 5, left (a)] but seeing-side fixation durations were significantly ***shorter*** than those of NV participants [*M:* 1.19 s vs. 2.17 s; β = 0.42, *SE* = 0.09, *t* = 4.79, *p* < 0.001; Figure 5, right (a)].

Participants were faster to first fixate a pedestrian when they fixated on cars from both sides compared to when they fixated on cross traffic only on one side [*M:* 2.41 s vs. 3.04 s; β = –0.12, *SE* = 0.06, *t* = –2.2, *p* = 0.028; Figure 5, left (b)], but pedestrian first-fixation times did not differ between situations with no cross-traffic fixations and only one side of cross-traffic fixations (M: 2.60 s vs. 3.04 s; β = –0.06, *SE* = 0.05, *t* = 1.35, *p* = 0.18). Participants spent more time looking at pedestrians when they did not fixate on any car compared to fixating on one side of cross traffic [*M:* 1.97 s vs. 1.82 s; β = –0.12, *SE* = 0.04, *t* = –2.86, *p* = 0.005; Figure 5, right (b)], but fixation durations did not differ between situations with only one side of cross-traffic fixations and both sides of cross-traffic fixations (*M:* 1.82 s vs. 1.87 s; β = 0.06, *SE* = 0.05, *t* = 1.20, *p* = 0.23).

### Safety Responses

In the last set of analyses, we were interested to see how participants responded to the crossing pedestrian when driving through an intersection. We evaluated the effects of subject group, number of cross-traffic cars fixated, and pedestrian approach direction (blind side or seeing side for HFL drivers, and right and left sides combined for NV drivers) on two parameters: the proportion of unsafe responses and the number of collisions.

#### Unsafe Responses to Crossing Pedestrians

**FIGURE 6:**
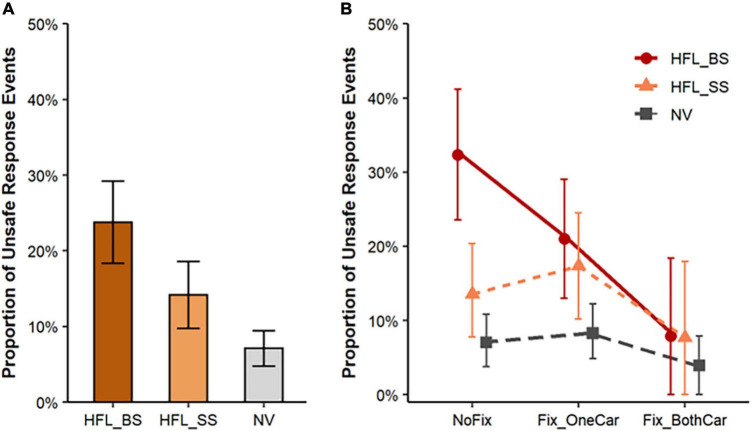
**(A)** Proportion of unsafe responses to crossing pedestrians when driving through the intersection: comparison between pedestrian approaching side for HFL (blind and seeing side) and NV groups. **(B)** The proportion of unsafe responses to crossing pedestrians showing a significant two-way interaction between direction of pedestrian approach (blind and seeing side for HFL) and cross-traffic car fixations (no fixation and fixated on one car). Error bars represent SEM.

HFL participants made significantly more unsafe responses to pedestrians crossing from their blind side than their seeing side (23.8% vs. 14.2%; β = –0.82, *SE* = 0.28, *z* = –2.98, *p* = 0.003; Figure 6, left) and twice as many unsafe responses on their seeing side than the NV group (14.2% vs. 7.1%; β = –1.44, *SE* = 0.82, *z* = –1.75, *p* = 0.079). The effects of fixating on zero, one or two sides of cross traffic differed between the HFL and NV groups (Figure 6, right). For HFL participants, unsafe response rates did not differ between the blind and seeing side when they looked at a car on only one side (Δ*M* = 3.7%: 21% vs. 17.3%). However, when they did not look at any cross-traffic cars, they had significantly more unsafe responses to pedestrians on the blind than the seeing side (Δ*M* = 18.8%: 32.4% vs. 13.6%; *Interaction*: β = 1.25, *SE* = 0.60, *z* = 2.09, *p* = 0.037; Figure 6, right). When HFL participants looked at both sides of cross-traffic cars, they had the fewest unsafe responses on both the blind and seeing sides and their unsafe response rates approached those of NV participants.

#### Number of Collisions

The HFL group had more collision events than the NV group (2.7% vs. 0.4%). Of 13 collision events among HFL participants, 8 collisions occurred with pedestrians approaching from the blind side. Of 13 collision events among HFL participants, 10 collisions occurred when participants did not fixate on cars. In contrast, none of the collision events occurred when participants fixated on both sides of cross-traffic cars.

## Discussion

Using a driving simulator, we quantified HFL and NV drivers’ gaze behaviors and pedestrian responses at intersections when different numbers of cross-traffic cars approached. HFL drivers exhibited compensatory behaviors by making more scans to their blind than seeing side (as previously reported, see Bowers, 2016), and also making larger gaze scans toward the blind side. Consistent with our hypotheses, blind-side gaze behaviors of HFL drivers differed between situations with and without the presence of a seeing-side car, and unsafe response rates were highest when there were no cross-traffic fixations.

Both HFL and NV participants looked at the majority of cross-traffic cars (>80%), regardless of whether there was a car from one or both sides. When HFL participants fixated on a car on one side only, first fixation times and fixation durations differed between Scenarios 2 and 3. In Scenario 2, where a cross-traffic car approached from only one side, HFL drivers took a similar amount of time to first fixate on blind-side and seeing-side cars, but spent about 0.4 s longer looking at cars approaching from their blind than seeing side. In contrast, in Scenario 3, where a cross-traffic car approached from both sides, when HFL participants fixated on just one of the cars, they were 0.5 s slower to make their first fixation on blind-side than seeing-side cars, but fixation durations did not differ between the two sides. The longer fixations on blind-side than seeing-side cross traffic when there was only one car approaching suggests compensatory behavior by HFL drivers, being more careful about cross traffic on their blind side since they could still use peripheral vision to monitor the seeing side while looking toward the blind side. However, in Scenario 3, where there was always a car on the seeing side, the presence of this seeing-side car seemed to cause HFL drivers to take longer to notice the blind-side car and spend less time fixating on it. Interestingly, when HFL participants fixated on both sides of cross-traffic cars, first fixation times and fixation durations did not differ for blind and seeing-side cars, and approached those of NV drivers.

When both HFL and NV participants made more scans, they were more likely to look at cross-traffic cars on both sides than one car or no cars. Furthermore, they made larger scans when they looked at cross traffic (at least one car) than when they did not fixate on any cars. It is possible that the cross traffic may have served as a reference point for HFL drivers when scanning to the blind side. A lack of guidance from peripheral vision on how far to scan has been suggested as a reason for blind-side scanning deficits of HFL drivers (Bowers et al., 2009, 2014; Swan et al., 2021b). Given the high fixation rates on cross-traffic cars, HFL drivers may have used the approaching car as a visual guide as to how far to scan, which may account for the larger blind side scans when cross-traffic cars were fixated.

Both groups were faster to make their first fixation on pedestrians when they looked at cross-traffic cars from both sides, and they spent longer looking at pedestrians when they did not fixate on any cross traffic. HFL drivers were about 1 s faster to first fixate and spent about 0.8 s longer fixating on blind than seeing-side pedestrians. The faster first fixations on blind-side pedestrians might seem counterintuitive, but is consistent with the finding that HFL participants made more first scans to their blind side (65% vs. 35% toward seeing side). Thus, they were able to detect pedestrians in their blind field sooner and fixate them for longer. Furthermore, the pedestrians standing at the zebra-marked intersection crosswalks subtended a relatively small eccentricity (about 4° to the left or right of the participant’s car heading direction) when cross-traffic cars were triggered to move. Therefore, the pedestrians were likely to fall within the range of the first scan to the blind side, which contributed to the probability of fixation after the first scan and the overall high fixation rate on blind side pedestrians (80%). Compared to NV participants, HFL participants took longer to first fixate (1.3 s longer) and spent less time (about 1 s less) fixating on seeing-side pedestrians. This may be because HFL participants spent more effort (longer fixations, more and larger gaze scans) on checking pedestrians and cross traffic on their blind side while using peripheral vision to monitor their seeing side.

Aligned with previous findings showing impaired detections and responses to hazards in the blind hemifield (Bowers et al., 2009; Papageorgiou et al., 2012; Bahnemann et al., 2015; Swan et al., 2021b), HFL participants had 1.6 times more unsafe responses to pedestrians on their blind than seeing side, and the majority (62%) of collisions occurred on their blind side. However, when HFL participants looked at cross-traffic cars on both sides, they exhibited the fewest unsafe responses (12% less than one side of car fixations and 23% less than no fixations) and no collisions occurred. There was an interaction between the side of the pedestrian approach and cross traffic fixations. Specifically, unsafe response rates to blind side pedestrians significantly decreased when HFL participants fixated on one side of cross traffic (from 32.4% for no cross-traffic fixations to 17.1%), but unsafe response rates to seeing side pedestrians did not differ between events when one or neither side of cross traffic was fixated. This finding suggests that fixating on cross traffic could improve HFL drivers’ safety responses to hazards on their blind side.

Even though HFL participants had overall more unsafe responses on both the blind and seeing sides than NV participants, their unsafe response rates did not differ between the blind and seeing sides and approached those of NV participants when they looked at cross traffic on both sides. In a similar vein, simulated driving studies of NV drivers found more traffic conflicts or collisions at the least complex intersections or when no oncoming traffic was present (Werneke and Vollrath, 2012; Kazazi et al., 2016). Thus monitoring cross traffic, especially on both sides, may increase safety awareness resulting in safer responses to other road users at intersections.

In this study, we used intersection scenarios where cross traffic and pedestrians followed normal traffic rules, which allowed participants to anticipate their behavior and respond to them as they would do when driving in real life. However, we used relatively simple scenarios where only one or no cars approached from each side. In future studies, we will extend our investigations to include different cross traffic flow densities and more diverse and complex intersection scenarios. It is possible that the effects of cross traffic fixations may differ in more complex scenarios. Driver’s expectations and perceptions for cross traffic and other vulnerable road users in the simulated driving environment may be different from real-world driving scenarios. Therefore, it will also be important to investigate the effects of cross traffic on real-world driving behaviors of individuals with HFL in a naturalistic driving study.

The presence of cross traffic encouraged more active scanning behaviors (greater gaze scan magnitudes and more gaze scans on both sides), which resulted in faster identification of a potential risk (pedestrians) and successful avoidance of blind-side collisions. Consistent with prior studies (Papageorgiou et al., 2012; Bahnemann et al., 2015; Alberti et al., 2017; Swan et al., 2021b), more active blind-side gaze scanning was associated with better blind-side detection performance and fewer collisions. In situations when HFL drivers scanned and fixated on cross traffic on both sides, their cross-traffic fixation behaviors, and pedestrian unsafe response rates, were most similar to those of NV drivers. However, when there was no car on the seeing side and they fixated a cross-traffic car on their blind side, then the cross-traffic fixation behaviors of HFL participants were the most different to those of NV drivers. Our scenario design also revealed unique insights about the effects of a seeing-side car; it took HFL drivers longer to first fixate a blind-side car and they fixated the car for less time when there was a car on the seeing side. In conclusion, the results from this study reinforce the need for drivers with HFL to proactively scan to both sides before entering an intersection. The presence of cross-traffic may encourage such scanning by acting as a reminder to scan and may also provide a reference point to guide blind-side scanning. The importance of actively scanning to check for and monitor cross traffic approaching from both sides should be emphasized in rehabilitation training for drivers with HFL.

## Data Availability Statement

The raw data supporting the conclusions of this article will be made available by the corresponding author upon reasonable request.

## Ethics Statement

The studies involving human participants were reviewed and approved by the Institutional Review Board (IRB) of Massachusetts Eye and Ear. The participants provided their written informed consent to participate in this study.

## Author Contributions

JX contributed to the programming, visualization and analysis of the data, and developed the driving simulation software and scenarios. All authors contributed to the manuscript and conceived of the experiment.

## Conflict of Interest

The authors declare that the research was conducted in the absence of any commercial or financial relationships that could be construed as a potential conflict of interest.

## Publisher’s Note

All claims expressed in this article are solely those of the authors and do not necessarily represent those of their affiliated organizations, or those of the publisher, the editors and the reviewers. Any product that may be evaluated in this article, or claim that may be made by its manufacturer, is not guaranteed or endorsed by the publisher.
