# Supplementary material for: Driving With Hemianopia X: Effects of Cross Traffic on Gaze Behaviors and Pedestrian Responses at Intersections
Source: Front Hum Neurosci. 2022 Jul 11;16:938140. doi: 10.3389/fnhum.2022.938140 (PMC9309302; doi:10.3389/fnhum.2022.938140)
Supplement: Supplementary file 1 [file Data_Sheet_1.pdf]

Supplementary Material

1.1 Supplementary Figure

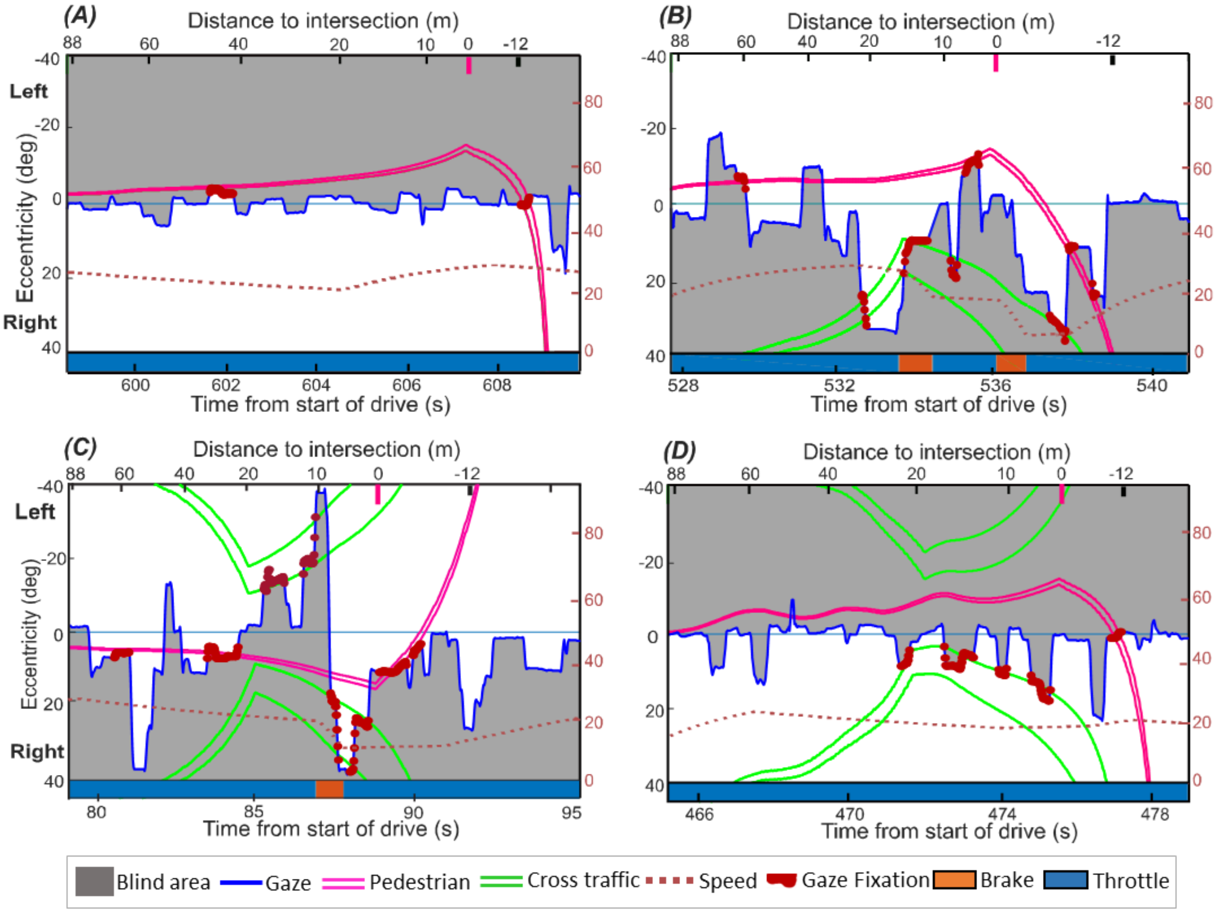

Supplementary Figure 1. Plots of lateral gaze (blue), pedestrian (pink), and cross traffic (green) position for critical event intersections for individual participants with HFL. Data are plotted from when the participant's car was 88.5 m away from the intersection to the time when the car passed the potential collision point. (For illustration purposes, eccentricity is limited to ± 40° on the y-axis. Cross-traffic cars started to move when the participant's car was 88.5 m from the intersection, but do not show up on the plots until their eccentricity is within ± 40°)

(A) Scenario 1-NoFix: There was no cross traffic in this event. The pedestrian was on the blind side (grey area) for the left HFL participant. The participant made their first fixation on the pedestrian at about 601.5s. At about 607s, the participant entered the intersection (pink tick mark at 0 m), and the pedestrian started running across the road. However, the participant noticed the crossing pedestrian very late, at about 608.5s, when the pedestrian was almost at the road center. In this example the participant did not press the brake pedal to slow down, and the response was classified as unsafe. Since there was no cross traffic in this event, it was classified as *NoFix*.

(B) Scenario 2-Fix_OneCar: When the right HFL participant drove toward the intersection, there was one car approaching from the blind side (right side) and the pedestrian was on the seeing side (left side). The participant made a blind side gaze scan (about 32°), scanned past then fixated on the right car while the car was approaching (at about 532.5s), then they braked to slow down soon afterward (at about 534s). While braking, the participant fixated on the car to make sure it totally stopped at the stop sign. Then they made a left gaze scan (about 17°) and noticed the pedestrian on the left side; they fixated on this pedestrian, braked again (at about 536s) and slowly drove across the intersection. This response was classified as safe. Since the participant fixated on the cross traffic, this event was categorized as *Fix_OneCar*.

(C) Scenario 3-Fix_BothCar*:* In this event, there was one car approaching from the left and one car from the right side at the same time. The right-side car and the pedestrian were on the right HFL participant's blind (right) side. The participant fixated on the pedestrian very early (at about 81s). Then a left gaze scan (about 18°) was made, allowing them to fixate on the seeing (left) side car (at about 85s). The participant then pressed the brake pedal to slow down (at about 87s). At almost the same time, the participant made a large scan (about 38°) to the blind (right) side and saw and then fixated on the right-side car. This blind (right) side scan brought the pedestrian into the visible portion of the visual field. They fixated and followed the crossing pedestrian for a while to make sure the pedestrian crossed the road. The participant then slowly drove through the intersection. The response was classified as safe. Since the participant fixated on both sides of cross traffic, this event was categorized as *Fix_BothCar*.

(D) Scenario 3-Fix_OneCar*:* This was another event with one car approaching from the left and one car from the right side at the same time. The left-side car and the pedestrian were on the left HFL participant's blind (left) side. During the whole event, the participant did not make any large gaze scans to the blind (left) side, so they did not notice the car on the left side. However, a gaze scan (about 10°) to the seeing (right) side allowed the participant to notice the right-side car (at about 471s) when the car had almost already reached the intersection. The participant fixated on this car a few times to make sure it came to a complete stop. Because there were no gaze scans to the blind (left) side, the participant did not notice the crossing pedestrian until they were already part of the way across the intersection (at about 477s). They did not brake or slow down during the whole event; the response was classified as unsafe. Since the participant only fixated on one side of cross traffic, this event was categorized as *Fix_OneCar*.
